# Colonoscopy Screening Among US Adults Aged 40 or Older With a Family History of Colorectal Cancer

**DOI:** 10.5888/pcd12.140533

**Published:** 2015-05-21

**Authors:** Meng-Han Tsai, Sudha Xirasagar, Yi-Jhen Li, Piet C. de Groen

**Affiliations:** Author Affiliations: Meng-Han Tsai, Yi-Jhen Li, University of South Carolina, Columbia, South Carolina; Piet C. de Groen, Mayo Clinic College of Medicine, Rochester, Minnesota.

## Abstract

**Introduction:**

Colonoscopy screening reduces colorectal cancer (CRC) incidence and mortality. CRC screening is recommended at age 50 for average-risk people. Screening of first-degree relatives of CRC patients is recommended to begin at age 40 or 10 years before the age at diagnosis of the youngest relative diagnosed with CRC. CRC incidence has increased recently among younger Americans while it has declined among older Americans. The objective of this study was to determine whether first-degree relatives of CRC patients are being screened according to recommended guidelines.

**Methods:**

We studied colonoscopy screening rates among the US population reporting a CRC family history using 2005 and 2010 National Health Interview Survey data.

**Results:**

Of 26,064 study-eligible respondents, 2,470 reported a CRC family history; of those with a family history, 45.6% had a colonoscopy (25.2% in 2005 and 65.8% 2010). The colonoscopy rate among first-degree relatives aged 40 to 49 in 2010 (38.3%) was about half that of first-degree relatives aged 50 or older (69.7%). First-degree relatives were nearly twice as likely as nonfirst-degree relatives to have a colonoscopy (adjusted odds ratio [AOR], 1.7; 95% confidence interval, 1.5–1.9), but those aged 40 to 49 were less likely to have a colonoscopy than those in older age groups (AOR, 2.6 for age 50–64; AOR, 3.6 for age ≥65). Interactions with age, insurance, and race/ethnicity were not significant. Having health insurance tripled the likelihood of screening.

**Conclusion:**

Despite a 5-fold increase in colonoscopy screening rates since 2005, rates among first-degree relatives younger than the conventional screening age have lagged. Screening promotion targeted to this group may halt the recent rising trend of CRC among younger Americans.

## Introduction

Colorectal cancer (CRC) is the second leading cancer in the United States; 132,700 new cases and 49,700 deaths are expected in 2015 (1–3). The 4.3% annual decline in incidence among adults aged 50 or older is marred by the concurrent 1.8% annual increase among adults younger than 50; an increase in CRC incidence of 28% to 46% is anticipated for this younger age group by 2030 (1,3). Younger CRC patients typically receive a diagnosis of more advanced disease and have poorer survival rates than older CRC patients, and they account for 6.5% of total CRC deaths (2,4).

About 30% of CRC patients report a family history of CRC: of those, 5% have one of the well-characterized inherited syndromes (eg, Lynch syndrome, familial adenomatous polyposis), and the remaining 25% are first-degree relatives of sporadic (nonhereditary) CRC patients. A first-degree relative is a biological parent, sibling, or child of a CRC patient (5–7). First-degree relatives have 2 to 3 times the risk of developing advanced adenomas and cancer than the general population. The risk increases as the relative’s age at diagnosis decreases and the number of relatives with CRC increases (8,9). About 23% of CRC patients younger than 45 years report a family history of CRC (10). Timely screening of first-degree relatives is therefore an important tool in decreasing rates of CRC.

Because colonoscopy allows for the removal of benign polyps that cause 75% to 80% of CRCs, colonoscopy screening can reduce CRC incidence by 83% and CRC mortality by 89% (11–14). The American College of Gastroenterology recommends first-degree relatives of CRC patients who received their cancer diagnosis before age 60 to begin colonoscopy screening at age 40 (13). The recent increase in CRC incidence among younger adults calls for greater attention to younger first-degree relatives (3,4). Studies on screening rates among first-degree relatives are dated, are limited to those aged 50 or older, or are single-center studies (15,16). Systematic reviews found low rates of colonoscopy screening among first-degree relatives (31%–40%) even though most guidelines emphasize the importance of colonoscopy screening for this higher-risk group (16). We conducted a population-based study of CRC screening among first-degree relatives younger (aged 40–49) than the conventional screening age of 50.

## Methods

### Study population

Pooled data from the 2005 and 2010 National Health Interview Survey (NHIS) were used. The NHIS is an annual, cross-sectional, nationally representative household survey that collects data on CRC every 5 years through the section “Family History and Cancer Screening.” The questions on cancer family history (Appendix) ask whether the respondent’s biological parent, sibling, or child ever had cancer and ask about the cancer type. The questions on CRC screening type and timing of the most recent screening test are consistent with the US Preventive Services Task Force (USPSTF) screening recommendations (12,13,17). Our study sample consisted of all screening-eligible respondents in the 2 surveys (Figure): all respondents aged 50 or older and all respondents aged 40 to 49 who reported a family history of CRC. Of the 36,575 respondents aged 40 or older in the 2 surveys, we excluded 10,511 respondents aged 40 to 49 who either reported not having a CRC family history (n = 3,517) or did not respond to the question (n = 6,994). Of 26,064 CRC screening–eligible respondents, 2,470 were first-degree relatives of CRC patients and 10,454 were nonfirst-degree relatives; we were unable to determine the family history of 13,140 respondents (aged 50 or older who did not respond to the family history question).

**Figure:**
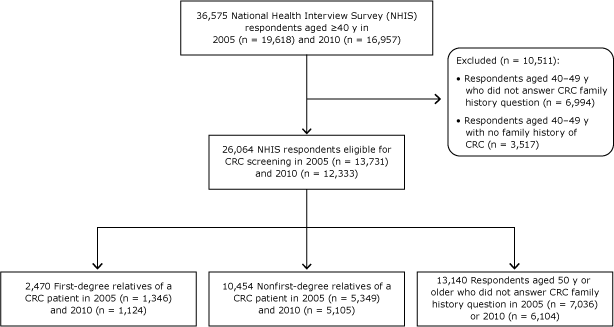
Study sample selected from respondents to the National Health Interview Surveys, 2005 and 2010. Abbreviation: CRC, colorectal cancer.

### Measures

Because colonoscopy is the most effective screening tool to prevent CRC and may be particularly important for first-degree relatives (11,13), we analyzed colonoscopy screening and any CRC screening. Per USPSTF recommendations, CRC screening completion was defined as the following: colonoscopy in the previous 10 years, a fecal blood test (FOBT or FIT) in the past year, sigmoidoscopy in the previous 5 years, or computed tomography (CT) colonography in the previous 5 years (12,13). Our key dependent variables of interest were colonoscopy completion (yes or no) and any CRC screening (yes or no). The key independent variables were family history of CRC (first-degree relative, nonfirst-degree relative, or no response to the question), age (40–49 y, 50–64 y, or ≥65 y), race/ethnicity (white, African American, Hispanic, or other) and health insurance (private; public, including Medicaid and Medicare; or uninsured). Survey year (2005 or 2010) was used to examine changes over time. We controlled for marital status and education level (18).

### Statistical analysis

We used χ^2^ tests to examine differences between subgroups separately for 2005 and 2010. Weighted logistic regression analyses of the pooled data were used to model the likelihood of colonoscopy screening (vs no colonoscopy) and any CRC screening (vs no screening). We studied associations with CRC family history, health insurance, age, sex, and race/ethnicity, controlling for marital status and education. Interactions of family history with age, sex, race/ethnicity, and health insurance were tested. We conducted weighted regression analyses to account for the stratification hierarchy and nonresponse bias and used a 2-sided test of significance at the .01 level as recommended by the National Center for Health Statistics. SAS v9.3 (SAS Institute Inc) was used.

## Results

The pooled sample consisted of 26,064 respondents (13,731 in 2005 and 12,333 in 2010). The colonoscopy rate was 49.1% in 2010, about 3 times the rate of 16.5% in 2005. Among first-degree relatives, 65.8% completed colonoscopy in 2010 and 25.2% in 2005, compared with 57.0% in 2010 and 19.0% in 2005 among non-first-degree relatives and 38.5% in 2010 and 12.7% in 2005 among nonrespondents (Table 1). Use of flexible sigmoidoscopy, FOBT, and CT colonography was negligible. Although the colonoscopy rate tripled from 2005 to 2010 among first-degree relatives aged 40 to 49 (Table 2), the rate among this younger group in 2010 (38.3%) was about half the rate (69.7%) among first-degree relatives aged 50 years or older (69.1% among adults aged 50–64 and 70.3% among those aged ≥65).

**Table 1:** Colorectal Cancer (CRC) Screening–Eligible Adult Respondents Aged 40 Years or Older, Classified By CRC Family History^a^, National Health Interview Survey 2005 and 2010 (n = 26,064)

Characteristic	2005 (n = 13,731)			2010 (n = 12,333)		
	Have a Family History,% (n = 1,346)	No Family History,% (n = 5,349)	No Response,% (n = 7,036)	Have a Family History,% (n = 1,124)	No Family History,% (n = 5,105)	No Response,% (n = 6,104)
**Sex^b^**						
Male	37.1	39.4	45.5	37.5	40.4	45.4
Female	62.9	60.6	54.5	62.5	59.6	54.6
**Age^b^, y**						
40–49	18.5	0	0	17.7	0	0
50–64	38.1	54.6	54.5	41.3	54.6	55.3
≥65	43.4	45.4	45.5	41.0	45.4	44.7
**Race/ethnicity^b^**						
White	85.5	87.0	74.7	83.1	83.6	71.1
African American	7.9	7.1	12.9	9.0	8.7	13.4
Hispanic	4.1	4.2	8.0	4.9	5.5	10.2
Other	2.4	1.8	4.5	2.9	2.2	5.3
**Insurance^b^**						
Private	44.9	43.8	41.9	46.3	41.2	40.0
Public	48.7	49.8	49.7	46.0	51.5	49.9
Uninsured	6.4	6.3	8.0	7.4	7.1	9.8
**CRC screening type^b^**						
Colonoscopy	25.2	19.0	12.7	65.8	57.0	38.5
Sigmoidoscopy	1.4	2.7	1.3	0.4	1.2	0.9
FOBT	0.5	0.5	0	1.7	3.2	2.6
CT colonography	NA	NA	NA	0.1	0	0.1
No screening	73.0	77.9	85.7	32.1	38.6	57.9

Abbreviations: CT, computed tomography; FOBT, fecal occult blood test; NA, not asked in the 2005 survey.

a Family history defined as self-reporting as a first-degree relative (biological parent, sibling, or child) of a CRC patient.

b *P* < .001 for all tests of difference between family history, no family history, and no response groups.

**Table 2:** Colorectal Cancer (CRC) Screening Status by Age Group and CRC Family History^a^, National Health Interview Survey 2005 and 2010 (n = 26,064)

Family History/Age Group	No.^b^	2005			2010		
		Colonoscopy	FS/FOBT/CT–C	No Screening	Colonoscopy	FS/FOBT/CT–C	No Screening
**Have a family history (n = 2,470)**							
40–49 y	251/196	12.7	1.6	85.7	38.3	3.1	58.7
50–64 y	524/470	21.4	1.3	77.3	69.1	1.7	29.1
≥65 y	571/458	32.0	2.5	65.5	70.3	2.2	27.5
**No family history (n = 10,454)**							
50–64 y	2,964/2,805	14.5	3.1	82.4	52.6	4.4	43.0
≥65 y	2,385/2,300	23.4	3.2	73.4	61.2	4.7	34.1
**No response (n = 13,140)**							
50–64 y	3,914/3,412	9.2	1.4	89.3	34.4	3.8	61.8
≥65 y	3,122/2,692	15.3	1.8	82.8	40.9	3.1	56.0

Abbreviations: CT–C, computed tomography colonography; FOBT, fecal occult blood test; FS, flexible sigmoidoscopy.

a Family history defined as self-reporting as a first-degree relative (biological parent, sibling, or child) of a CRC patient.

b Total respondents in the category in 2005 and 2010, respectively.

The likelihood of having a colonoscopy versus not having a colonoscopy was 5 times higher in 2010 than in 2005 after adjustment for age, sex, race/ethnicity, health insurance, and other covariates (adjusted odds ratio [AOR], 5.4; 95% confidence interval [CI], 5.0–5.8) (Table 3). First-degree relatives were 70% more likely than nonfirst-degree relatives to have a colonoscopy (AOR, 1.7; 95% CI, 1.5–1.9). Nonrespondents were about half as likely as nonfirst-degree relatives to have a colonoscopy. The likelihood of colonoscopy among first-degree relatives aged 40 to 49 was about one-third that of the older age groups (AOR, 2.6 for those aged 50–64; AOR, 3.6 for those aged ≥65). Respondents who had private or public health insurance were 3 times as likely as those who were not insured to have a colonoscopy (AOR, 3.3 for private insurance; AOR, 3.4 for public insurance). African Americans and whites were equally likely to have a colonoscopy. Interactions of first-degree relative status with age, health insurance, and race/ethnicity were not significant. The results did not change when data on all screening tests were combined.

**Table 3:** Adjusted Likelihood of Colonoscopy Among US Adults Aged 40 or Older With a Family History of Colorectal Cancer (CRC)^a^, National Health Interview Survey 2005 and 2010 (n = 26,064)^b^

Category	Colonoscopy (vs No Colonoscopy), Odds Ratio (95% Confidence Interval)
**Year**	
2005	1.0 [Reference]
2010	5.4 (5.0–5.8)
**Sex**	
Male	1.0 (0.9–1.1)
Female	1.0 [Reference]
**Age**	
40–49^b^	1.0 [Reference]
50–64	2.6 (2.0–3.3)
≥65	3.6 (2.7–4.7)
**Race/ethnicity**	
White	1.0 [Reference]
African American	1.0 (0.9–1.1)
Hispanic	0.8 (0.7–1.0)
Other	0.7 (0.6–0.8)
**Family history of CRC**	
Have a family history	1.7 (1.5–1.9)
No family history	1.0 [Reference]
No response	0.5 (0.5–0.6)
**Insurance**	
Private	3.3 (2.8–3.9)
Public	3.4 (2.8–4.1)
Uninsured	1.0 [Reference]

a Family history defined as self-reporting as a first-degree relative (biological parent, sibling, or child) of a CRC patient.

b Additionally adjusted for marital status and education. Income was not significant and was excluded from the model.

## Discussion

The key finding of this study is that first-degree relatives younger than the conventional screening age of 50 were less likely than adults aged 50 or older to have had a colonoscopy. Although studies document screening rates among first-degree relatives of inherited CRC syndromes, few studies are available on first-degree relatives of sporadic CRC patients, even though they are the largest higher-risk subgroup in the population. Our study shows the need for increasing screening rates in this subgroup, particularly first-degree relatives younger than 50 because of the recent increase in CRC among American adults in this age group.

The low colonoscopy rate among younger first-degree relatives (40%) observed in our study should be viewed in light of the multifold increase in colonoscopy rates between 2005 and 2010 (69.7%) among adults in the widely publicized screening age group. Studies of first-degree relatives of sporadic CRC patients either focused on first-degree relatives aged 50 or older or used mixed-age samples without distinguishing younger first-degree relatives. Our other study findings are also consistent with those of earlier studies (19-22). Among the population aged 50 or older, colonoscopy rates were higher among first-degree relatives than among nonfirst-degree relatives. A recent study using NHIS 2010 data on adults aged 50 or older reported colonoscopy rates of 72.3% among first-degree relatives and 53.5% among nonfirst-degree relatives, similar to our finding (19). A study based on NHIS 2000 data on adults aged 41 to 75 reported colonoscopy rates of 27.8% among first-degree relatives and 7.7% among nonfirst-degree relatives (20). Only 1 population-based study using NHIS 2000 data on younger first-degree relatives is available: although it did not distinguish among screening types, it reported that 15.8% of men and 8.9% of women aged 40 to 49 had a CRC screening test (22). One meta-analysis of 7 studies reported a pooled colonoscopy rate of 40% among all first-degree relatives aged 40 or older; no study in the analysis included recent data (23).

Our findings are also consistent with the findings of single-center studies. A practice-based patient survey in 2004 showed a colonoscopy rate of 29.6% among first-degree relatives younger than 50 and a rate of 76% among first-degree relatives aged 50 or older. Only 39% of first-degree relatives younger than 50 had ever been asked by their physician about a CRC family history, and almost half (46%) believed that screening should begin at age 50 (24). Another study reported that the lack of physician recommendation was the single most important reason that first-degree relatives younger than 50 years had not undergone colonoscopy screening (25). Lack of awareness among first-degree relatives of the need for early screening and lack of physician recommendation appear to be major reasons for the low screening rates among first-degree relatives younger than 50. Screening education may have a greater effect among first-degree relatives because of their personal exposure to CRC through family members.

Consistent with prior studies, we found that having health insurance increased the likelihood of colonoscopy screening (19,23,26,27). Colonoscopy screening rates among Medicare enrollees increased after 2001, when colonoscopy coverage was launched, from a mean quarterly rate of 285 per 100,000 beneficiaries during 1992–1997 to 1,919 per 100,000 beneficiaries during 2001–2002 (28). The Affordable Care Act (ACA) now requires first-dollar coverage of preventive services, including colonoscopy, a provision that was not in force during the NHIS 2010 survey. Screening promotion among younger first-degree relatives in the ACA environment has a better chance of increasing screening rates than in the pre-ACA environment, although we would expect some attenuation of effect because of the general tendency of younger adults not to avail themselves of preventive health services and because grandfathered health plans are not required to conform to ACA provisions (4).

Our study has several limitations. One is response-rate bias: half of the sample did not answer the family history question, and they were systematically different from the half that did answer the question: they were half as likely as nonfirst-degree relatives to have undergone colonoscopy screening. Another limitation is that data were self-reported (ie, data were not extracted from medical records), which may have resulted in overestimation of screening rates (19,29). Finally, imbalanced cell sizes of the family history variable (2,470 vs 10,454) may limit the accuracy of odds ratio estimates.

Despite these limitations, our study is important in highlighting that first-degree relatives aged 40 to 49 of CRC patients are an undertargeted (and potentially rewarding) group for focused promotion of CRC prevention. Screening promotion should target both physicians and patients: alerting primary care physicians to engage younger patients in learning about a potential CRC family history and educating CRC patients to alert their first-degree relatives to initiate screening discussions with their physicians. Our recent report of an 83% reduction in CRC incidence and an 89% reduction in CRC mortality after screening colonoscopies should boost enthusiasm for colonoscopy screening among both patients and physicians (11). Coupled with the ACA provisions requiring coverage of screening procedures, such efforts can help arrest the increase in CRC among Americans younger than 50 years (1,10).

## Appendix National Health Interview Survey (NHIS) Questions on Colorectal Cancer Family History and Screening Tests, 2005 and 2010

The survey questions on colorectal cancer family history and screening are located in the sample adults cancer file. Questions are similar in both years. The original questions used to create the study variables are the following:

**Table:**

Question	Answer
What kind of cancer did your father have . . . colon?	1, Mentioned; 2, Not mentioned; 7, Refused; 8, Not ascertained; 9, Don’t know
What kind of cancer did your father have . . . rectum?	
What kind of cancer did your mother have . . . colon?	
What kind of cancer did your mother have . . . rectum?	
What kind of cancer did your (brother/brothers) have . . . colon?	
What kind of cancer did your (brother/brothers) have . . . rectum?	
What kind of cancer did your (brother/brothers) have . . . rectum?	
What kind of cancer did your (sister/sisters) have . . . colon?	
What kind of cancer did your (sister/sisters) have . . . rectum?	
What kind of cancer did your (son/sons) have . . . colon?	
What kind of cancer did your (son/sons) have . . . rectum?	
What kind of cancer did your (daughter/daughters) have . . . colon?	
What kind of cancer did your (daughter/daughters) have . . . rectum?	
**NHIS 2010**	
Most recent sigmoidoscopy, time categories (using 2005 method)	1, A year ago or less; 2, More than 1 year but not more than 2 years; 3, More than 2 years but not more than 3 years; 4, More than 3 years but not more than 5 years; 5, More than 5 years but not more than 10 years; 6, Over 10 years ago; 7, Refused; 8, Not ascertained; 9, Don't know
Most recent home blood stool test, time categories (using 2005 method)	
Most recent CT colonography or virtual colonoscopy, time categories	
**NHIS 2005**	
Was this MOST RECENT exam a sigmoidoscopy, colonoscopy, proctoscopy or something else?	1, Sigmoidoscopy; 2, Colonoscopy; 3, Proctoscopy; 4, Something else; 7, Refused; 8, Not ascertained; 9, Don’t know
Most recent colorectal exam, time categories	1, A year ago or less; 2, More than 1 year but not more than 2 years; 3, More than 2 years but not more than 3 years; 4, More than 3 years but not more than 5 years; 5, More than 5 years but not more than 10 years; 6, Over 10 years ago; 7, Refused; 8, Not ascertained; 9, Don’t know
Most recent office blood stool test, time categories	
